# *TPM1*-p.E181K mutation suppresses CaMKII/HDAC4 signaling pathway leading to pediatric restrictive cardiomyopathy

**DOI:** 10.3389/fgene.2025.1654907

**Published:** 2026-01-07

**Authors:** Jia Fu, Jing Zhang, Youxian Zhang, Dongming Sun, Kun Xia, Ruigeng Wang, Xiaoyuan Feng, Aiguo Zhai, Yufeng Huang, Xiaobin Li, Wenjun Yu, Yong Zhang

**Affiliations:** 1 Department of Cardiology, Wuhan Children’s Hospital (Wuhan Maternal and Child Healthcare Hospital), Tongji Medical College, Huazhong University of Science and Technology, Wuhan, China; 2 Department of General Surgery, Hubei Provincial Hospital of Integrated Chinese and Western Medicine, Wuhan, China; 3 Wuhan Children’s Hospital (Wuhan Maternal and Child Healthcare Hospital), Tongji Medical College, Huazhong University of Science and Technology, Wuhan, China; 4 College of Food Science and Engineering, Shannxi University of Science and Technology, Xi’an, China; 5 Department of Cardiovascular Surgery, Zhongnan Hospital of Wuhan University, Wuhan, China; 6 Wuhan Clinical Research Center for Minimally Invasive Treatment of Structural Heart Disease, Wuhan, China

**Keywords:** CaMKII/HDAC4, molecular mechanisms, pediatric cardiology, restrictive cardiomyopathy, TPM1-p.E181K

## Abstract

**Background:**

This study aims to elucidate the pathogenicity of the *TPM1* mutation (NM_001018005.2:c.541G>A, p. Glu181Lys) in restrictive cardiomyopathy (RCM), establish its ACMG pathogenicity classification, and report for the first time its association with sporadic RCM and underlying molecular mechanisms. The research focuses on delineating how this mutation triggers myocardial pathology via disruption of the CaMKII/HDAC4 signaling pathway.

**Methods:**

Protein 3D modeling predicted structural alterations induced by the mutation. *TPM1*-wild-type (WT) and mutant (p.E181K) plasmids were transfected into AC16 cardiomyocyte cell lines. Quantitative PCR (qPCR) and Western blotting (WB) analyzed gene/protein expression levels. Intracellular calcium transients were detected using Rhod-2 AM fluorescent probes. F-actin cytoskeletal reorganization was assessed by Phalloidin-488 staining. Phosphorylation status of key CaMKII/HDAC4 pathway components and troponin (Tn) activity were evaluated to define functional mechanisms.

**Results:**

Bioinformatic analysis revealed disruption of hydrogen bonding and electrostatic potential at the mutation site. *TPM1*-p.E181K did not alter overall protein expression or mitochondrial activity but significantly suppressed intracellular Ca^2+^ transients and inhibited CaMKII/HDAC4 phosphorylation. Impaired troponin activity and abnormal cardiomyocyte contractility were observed.

**Conclusion:**

This study establishes a novel link between TPM1-p.E181K and sporadic RCM. We demonstrate that its pathogenesis is mediated through a cascade of events: calcium dyshomeostasis leads to the suppression of CaMKII/HDAC4 phosphorylation, which subsequently causes sarcomere structural disruption, and ultimately results in myocardial hypercontractility. This identified signaling axis may represent a promising therapeutic target for RCM.

## 1 Introduction

Restrictive cardiomyopathy (RCM) is a rare form of cardiomyopathy characterized by impaired diastolic function with preserved systolic function, left atrial or biatrial enlargement, elevated atrial pressure, and normal or near-normal ventricular wall thickness (Dadson et al., 2017; Elliott et al., 2008). Although RCM accounts for a relatively low proportion of childhood cardiomyopathies (approximately 2%–5%), its prognosis is extremely poor, with a 2-year mortality rate as high as 50% (including sudden cardiac death) in pediatric patients, imposing a substantial burden on families and society (Webber et al., 2012). RCM exhibits significant genetic heterogeneity, with over 19 disease-associated genes currently reported (Chintanaphol et al., 2022). These genes primarily encode two categories of proteins: sarcomeric proteins (e.g., *TNNI3*, *TNNT2*, *TNNC1*, *TPM1*, *MYH7*, *MYBPC3*, *MYPN*) and non-sarcomeric proteins (e.g., *TTN*, *DES*, *FLNC*, *LMNA*, *CRYAB*, *BAG3*, *ABCC9*). Notably, while mutations in *TPM1* (encoding the α-tropomyosin chain) have been well-established in hypertrophic cardiomyopathy (HCM) and dilated cardiomyopathy (DCM) (Rivenes et al., 2000; Lipshultz et al., 2019), definitive pathogenic evidence linking it to RCM remains exceptionally rare, and the specific molecular mechanisms are unclear. This knowledge gap underscores the critical need to explore the pathogenic mechanisms of rare genetic variants in RCM, which is essential for precise diagnosis and targeted therapeutic development.

In an 11-year-old boy with severe sporadic RCM (negative family history), we identified a *de novo* missense mutation (*TPM1* NM_001018005.2: c.541G>A, p. Glu181Lys). This case report has been published in the Chinese Journal of Pediatrics (Fu et al., 2022). The mutation site (Glu181) exhibits high cross-species conservation, suggesting its critical role in TPM1 protein function. This finding strongly implicated TPM1-p.Glu181Lys in sporadic RCM pathogenesis; however, its definitive pathogenicity and molecular mechanisms required experimental validation. Therefore, the objectives of this study were to establish the functional association between TPM1-p.Glu181Lys and the RCM phenotype and to elucidate its molecular mechanisms in inducing cardiomyopathy. Given TPM1’s central role in regulating myofilament calcium sensitivity, we specifically investigated whether this mutation disrupted calcium homeostasis, thereby impairing key signaling pathways (e.g., CaMKII/HDAC4), which ultimately led to sarcomeric structural and functional abnormalities (e.g., hypercontractility, diastolic dysfunction). This study aimed to provide *in vitro* functional evidence supporting the pathogenicity of TPM1-p.Glu181Lys and to evaluate its clinical significance according to the ACMG guidelines.

This study is the first to systematically explore and reveal the mechanistic link between *TPM1* mutations and RCM pathogenesis (particularly via the CaMKII/HDAC4 axis), offering new insights into the genetic etiology of RCM and identifying potential therapeutic targets.

## 2 Materials and methods

### 2.1 Study subjects and ethical compliance

This study was approved by the Medical Ethics Committee of Wuhan Children’s Hospital (Approval No.: 2024R043-E01), and written informed consent was obtained from both the patient’s legal guardians and his parents. The study subject was an 11-year-old male RCM patient who met the diagnostic criteria below and had no family history of cardiomyopathy.

### 2.2 Diagnostic criteria for restrictive cardiomyopathy

RCM diagnosis strictly adhered to the 2019 American Heart Association (AHA) classification criteria for cardiomyopathies (Rivenes et al., 2000; Lipshultz et al., 2019) and the American Society of Echocardiography (ASE) guidelines for diastolic function assessment (Nagueh et al., 2016).

### 2.3 TPM1 mutation analysis

Genomic DNA was extracted from peripheral blood samples of the proband, parents, and sister. Whole-exome sequencing (WES) was performed using the MGISEQ-2000 platform (150-bp paired-end reads, mean depth >100×). Variants with population frequency >0.1% (gnomAD/1000 Genomes) were filtered out. Variant interpretation followed the ACMG/AMP 2015 guidelines using InterVar v2.0.

### 2.4 Plasmid construction

The wild-type TPM1 gene (NM_001018005.2) was cloned into the pCMV-3XFlag-Neo (EGFP) vector (Provided by Bioeagle Biotech Company) using EcoRI/BamHI restriction sites. Site-directed mutagenesis to introduce the c.541G>A variant was performed with the QuikChange II XL Kit using the following primers:

Forward: 5′-CTG​GAA​CGT​GCA​GAG​AAG​CGG​GCT​GAG​CTC-3′

Reverse: 5′-GAG​CTC​AGC​CCG​CTT​CTC​TGC​ACG​TTC​CAG-3′

Successful mutagenesis was confirmed by bidirectional Sanger sequencing with T7 promoter and terminator primers.

### 2.5 Cell culture and transfection

AC16 human cardiomyocytes were cultured in DMEM/F12 medium supplemented with 15 mM HEPES, 10% fetal bovine serum, and 1% penicillin/streptomycin at 37 °C under 5% CO_2_. For transfection, 5 × 10^5^ cells/well were seeded in 6-well plates. Transfection complexes were prepared at a DNA (µg): Lipofectamine 2000 (µL) ratio of 1:2. After 6 h, the medium was replaced with complete growth medium, followed by 48-h incubation.

### 2.6 Gene expression analysis

Total RNA was isolated using TRIzol reagent, followed by cDNA synthesis with the PrimeScript RT Reagent Kit. Quantitative PCR (qPCR) was performed on the QuantStudio 6 Flex Real-Time PCR System using UltraSYBR Green PCR SuperMix and gene-specific primers (forward: 5′-AGT​CGA​GCC​CAA​AAA​GAT​GA-3′; reverse: 5′-CCT​GAG​CCT​CCA​GTG​ACT​TC-3′), with the amplification cycle performed according to the manufacturer’s instructions.

### 2.7 Mitochondrial function and calcium signaling assays

ATP levels were quantified using the ATP Assay Kit (Jiancheng Bio, Cat# A095-1) via phosphomolybdate colorimetry. Absorbance at 636 nm was measured with a microplate reader (Molecular Devices, SpectraMax CMax Plus), and ATP content was calculated as µmol/g protein. For calcium detection, transfected cells were incubated with 5 µM Rhod-2 AM in the dark at 37 °C for 30 min. Fluorescent images were captured using an inverted fluorescence microscope (Olympus IX51), and fluorescence intensity was quantified with ImageJ v1.53.

### 2.8 Phosphorylation analysis of signaling pathways

Transfected AC16 cells were lysed in RIPA buffer (Aspen, Cat# AS1004). Proteins (20 µg) were resolved on 10% SDS-PAGE (100V, 120 min), transferred to Polyvinylidene fluoride (PVDF) membranes (300mA, 120 min), and blocked with 5% skim milk. Membranes were incubated with primary antibodies: p-CaMKII(Thr286) (1:1000, abcam, Cat# ab320638), CaMKII (1:1000, Cell Signaling Technology [CST], Cat# 50049), p-HDAC4(Ser632) (1:500, abcam, Cat# ab39408), HDAC4 (1:1000, abcam, Cat# ab39408), and GAPDH (1:10000, abcam, Cat# ab181602). After washing, the membranes were incubated with HRP-conjugated secondary antibodies: HRP-Goat anti-Rabbit (1:10000, ASPEN, Cat# AS1107) and HRP-Goat anti-Mouse (1:10000, ASPEN, Cat# AS1106). Protein expression was quantified using AlphaEaseFC software.

### 2.9 Morphological analysis

After transfection, cells were fixed with 4% paraformaldehyde for 20 min and permeabilized with 0.1% Triton X-100 for 10 min. Subsequently, the cells were stained with Phalloidin-488 (1:200 dilution) for 60 min to visualize F-actin, followed by DAPI (1 μg/mL) for 5 min to label the nuclei. Finally, the samples were imaged using an inverted microscope (Olympus IX51) to evaluate sarcomeric organization and structural integrity.

### 2.10 Statistical analysis

Data are expressed as mean ± standard deviation (SD). Intergroup comparisons used Student’s t-test. Analyses were performed in SPSS v20.0; figures were generated with GraphPad Prism v9.0. P ≤ 0.05 was considered statistically significant.

## 3 Results

### 3.1 RCM Pedigree and Proband Clinical Phenotype

**FIGURE 1:**
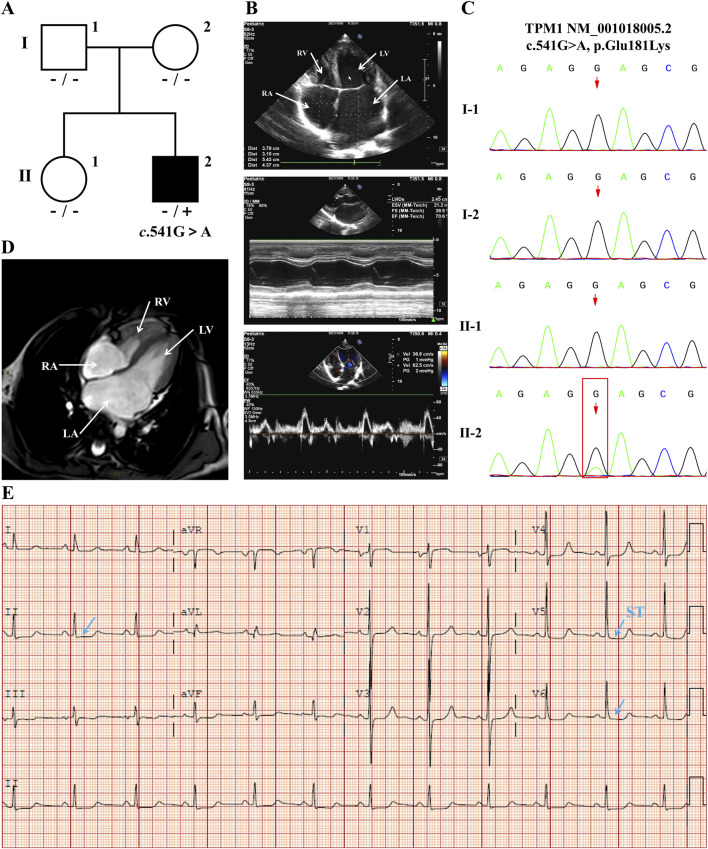
Integrated Analysis of RCM Pedigree and Proband Clinical Phenotype **(A)** Pedigree analysis identified the proband (II-2) as heterozygous for the *TPM*1 c.541G>A mutation, while parents (I-1 and I-2) and sister (II-1) showed the wild-type sequence. **(B)** Echocardiography revealed: biatrial enlargement (apical four-chamber view, LA marked); preserved left ventricular systolic function by M-mode (EF = 70.6%); and diastolic dysfunction evidenced by mitral inflow patterns (E/A = 1.7; E/e’ = 11, LAVI = 40 mL/m^2^). **(C)** Sanger sequencing confirmed this heterozygosity in the proband (red box indicating c.541G>A site) versus parental wild-type genotypes. **(D)** Cardiac MRI (four-chamber view) demonstrated biatrial enlargement with predominant left atrial dilation. **(E)** Electrocardiography showed sinus rhythm with QT prolongation (QTc: 477 ms) and ST-segment changes (arrows). (Imaging parameters: Philips EPIQ5 ultrasound (S8-3 probe); ECG: Philips PagewriterTC (25 mm/s); MRI: Philips Ingenia 3.0T CX).

The 11-year-old male proband presented with short stature (height: 125 cm, <3rd percentile). Pedigree analysis (Figure 1A) identified a heterozygous *TPM1* c.541G>A (p.Glu181Lys) mutation in the proband (II-1), while Sanger sequencing confirmed wild-type genotypes in both parents (I-1, I-2) and sister (II-2), establishing the mutation’s *de novo* status (Figure 1C). Clinical evaluation revealed significant cardiac abnormalities: cardiac MRI documented biatrial enlargement (Figure 1D); echocardiography demonstrated marked left atrial dilation with mild right atrial enlargement (Figure 1B); electrocardiography showed sinus rhythm with ST-segment depression >0.15 mV in leads V4-V6 and QTc prolongation to 477 ms (Figure 1E). Diastolic dysfunction was evidenced by a mitral inflow E/A ratio of 1.7. The patient exhibited no respiratory symptoms (fever, cough, vomiting, or diarrhea) or exertional cardiorespiratory complaints (dyspnea or cyanosis). This phenotypic profile—characterized by biatrial dilation and diastolic impairment—fulfilled diagnostic criteria for sporadic restrictive cardiomyopathy (RCM).

### 3.2 Structural impact of *TPM1*-p.E181K mutation

**FIGURE 2:**
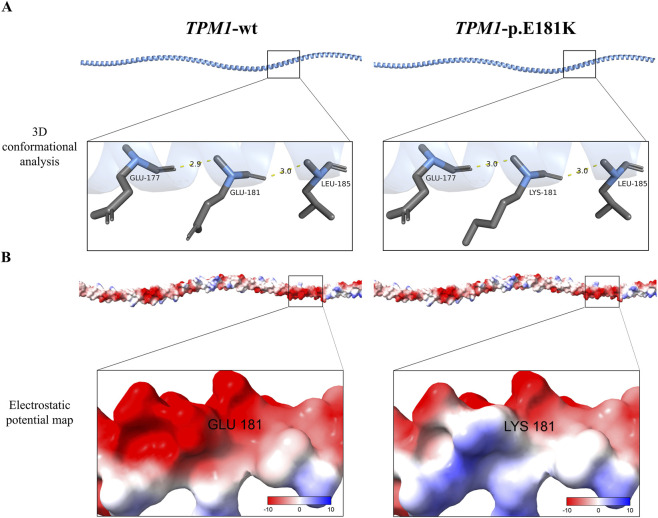
Structural and electrostatic alterations induced by *TPM1*-p.E181K **(A)** Conformational changes at the mutation site (position 181) extend the backbone hydrogen bond distance between Lys^181^ and Glu^177^ from 2.9 Å in wild-type to 3.0 Å (Δ = 0.1 Å), while maintaining the Leu^185^ hydrogen bond distance (3.0 Å). **(B)** APBS-based surface electrostatic potential mapping demonstrates a reversal of charge polarity, with the negative potential (red) changing to positive potential (blue).

Multidimensional computational analyses assessed the effects of the *TPM1* p. Glu181Lys mutation on protein structure. AlphaFold2 modeling coupled with PyMOL structural analysis demonstrated that this mutation induces remodeling of critical hydrogen bond networks (Figure 2A). This conformational perturbation potentially destabilizes the α-helical integrity within the tropomyosin functional domain (residues 170–190). SOPMA secondary structure prediction further confirmed increased structural rigidity: the alpha helix (Hh) proportion rose from 98.59% (wild-type) to 98.94%, while random coil (Cc) content decreased from 1.41% (wild-type) to 1.06%, indicating abnormal flexibility due to altered α-helix-to-coil ratios. Surface electrostatic potential analysis (ChimeraX/APBS) revealed charge polarity reversal: wild-type Glu181 exhibited strong negative charge (−8 kT/e, red regions), whereas mutant Lys181 displayed positive potential (+6 kT/e, blue regions), disrupting electrostatic complementarity at the actin-binding interface (Figure 2B).

### 3.3 Effect of *TPM1*-p.E181K on basal expression and calcium homeostasis

**FIGURE 3:**
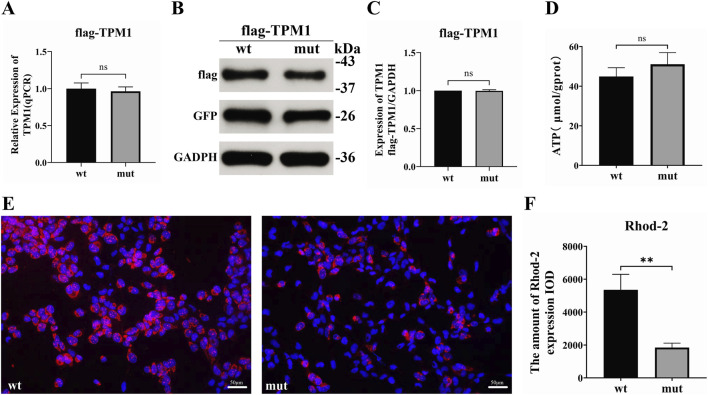
Impact of *TPM*1-p.E181K mutation on basal cellular function **(A)** Relative TPM1 mRNA expression (qPCR, n = 6). **(B)** Representative Western blotting bands. **(C)** Protein quantification by grayscale analysis. **(D)** Intracellular ATP concentration (μmol/g protein). **(E)** Rhod-2 staining demonstrating diminished calcium signals (pseudocolored red; scale bar = 50 μm). **(F)** Quantitative analysis of calcium fluorescence intensity expressed as Integrated Optical Density (IOD), a measure of total fluorescence signal within the measured area. Data are presented as arbitrary units (Wild-type: 5352.04 ± 943.84; Mutant: 1844.7 ± 271.06).

qPCR analysis revealed comparable *TPM1* mRNA levels in AC16 cardiomyocytes transfected with TPM1-p.Glu181Lys versus wild-type (98.5% ± 8.1%; t = 0.43, P = 0.67, n = 3) (Figure 3A). Western blot quantification confirmed no significant difference in protein expression (relative grayscale: 99.7% ± 2.1%; t = 0.38, P = 0.72, n = 3) (Figures 3B,C). Colorimetric ATP measurements showed no statistical difference between mutant and wild-type cells (WT: 44.9 ± 4.4 μmol/g prot; Mut: 51.1 ± 5.9 μmol/g prot; t = 1.45, P = 0.22, n = 3) (Figure 3D), indicating no direct impact on energetic metabolism homeostasis. Rhod-2 AM fluorescent probe detection demonstrated significantly reduced intracellular Ca^2+^ concentration in mutant cells versus wild-type (t = 6.17, P < 0.0035, n = 50 cells/group) (Figures 3E,F). Fluorescence microscopy visibly demonstrated diminished red fluorescence intensity in mutant cells (Figure 3E), a phenotype concordant with pathological calcium dysregulation in restrictive cardiomyopathy.

### 3.4 Suppression of CaMKII/HDAC4 phosphorylation cascade by *TPM1*-p.E181K

**FIGURE 4:**
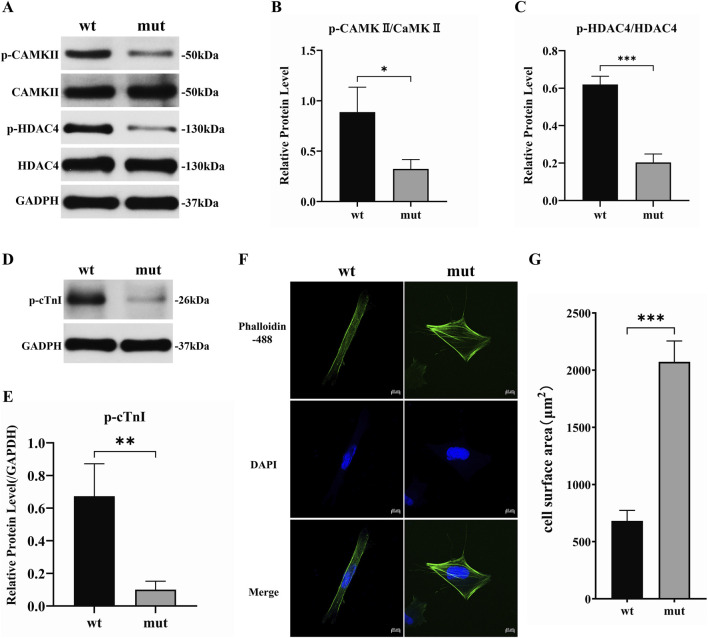
TPM1-p.E181K suppresses phosphorylation signaling and induces sarcomeric remodeling **(A)** Western blot showing reduced p-CaMKII and p-HDAC4 expression. **(B)** p-CaMKII/CaMKII grayscale quantification (α-subunit: 50 kDa; loading control: β-actin). **(C)** p-HDAC4/HDAC4 quantification (α-subunit: 130 kDa). **(D)** Western blot demonstrating decreased p-cTnI expression. **(E)** p-cTnI grayscale quantification (α-subunit: 24 kDa; loading control: β-actin). **(F)** Immunofluorescence co-staining: F-actin (Phalloidin-488, green), nuclei (DAPI, blue) (scale bar: 20 μm). **(G)** Cell surface area quantification (WT: 681.3 ± 92.0 μm^2^ vs. Mut: 2073.6 ± 182.1 μm^2^).

Western blot quantification revealed significantly reduced phosphorylation levels of CaMKII in mutant versus wild-type cells (P = 0.021), with an even more pronounced reduction in HDAC4 phosphorylation (P = 0.0003) (Figures 4A–C). Mechanistically, diminished calcium signaling (Figure 3E) impaired CaMKII kinase activity, thereby suppressing HDAC4 phosphorylation.

### 3.5 Impaired troponin phosphorylation validates contractile dysregulation

Phosphorylated troponin I (p-TnI) expression decreased by 57.3% ± 11.9% in mutant cells (relative levels: WT: 0.67 ± 0.07 vs. Mut: 1.00 ± 0.12; t = 4.83, P = 0.0084, n = 6) (Figures 4D,E), indicating that the TPM1 mutation disrupts myofilament contractile regulation via the CaMKII-TnI signaling axis.

### 3.6 Morphological evidence of pathological remodeling in cardiomyocytes

Phalloidin-488/DAPI confocal imaging demonstrated increased cell surface area in mutants. Quantitative analysis confirmed a mean increase of 1392 ± 117.7 μm^2^ (t = 11.83, P = 0.0003, n = 50 cells/group) (Figures 4F,G).

## 4 Discussion

Tropomyosin (TM), an actin-binding protein composed of two α-helical chains forming coiled-coil structures, serves as a critical regulator of muscle contraction. It exists as multiple isoforms across eukaryotic cells and aligns with actin filaments to modulate actin-actin interactions. In mammals, 4 TM genes (*TPM1*, *TPM2*, *TPM3*, *TPM4*) encode over 20 TM isoforms (Perry, 2001). The *TPM1*-p.E181K missense mutation localizes to exon 5 of chromosome 15 (residue 181/284aa) within the conserved tropomyosin functional domain—a key actin-binding region. Therefore, mutations in the *TPM1* gene exclusively cause cardiomyopathy without associated clinical myopathy—despite its expression in skeletal muscle—likely mediated by C-terminal alternative splicing of tissue-specific isoforms (Redwood and Robinson, 2013). Pathogenic TPM1 mutations account for 1%–2% of dilated cardiomyopathy (DCM) cases (Hershberger et al., 2010; Shen et al., 2022), 5% of hypertrophic cardiomyopathy (HCM) cases (Watkins et al., 1995; Coviello et al., 1997; Tardiff, 2005), and 3% of restrictive cardiomyopathy (RCM) cases, based on limited cohort evidence (Gallego-Delgado et al., 2016).

Genetic sequencing of a pediatric RCM case revealed the *de novo* TPM1-p.E181K mutation. Pathogenicity predictions unanimously indicated deleterious effects: SIFT (D), PolyPhen2_HDIV (D), PolyPhen2_HVAR (D), REVEL (0.844). Structural modeling (Figure 2) further supported functional disruption. The variant was absent from ClinVar, and its *de novo* origin was confirmed by parental sequencing. Per ACMG guidelines (Richards et al., 2015), this variant is classified as Likely Pathogenic.

Functionally, the TPM1-p.E181K mutation significantly suppressed the phosphorylation of both CaMKII (P < 0.05) and HDAC4 (P < 0.001) (Figures 4A–C). We propose that calcium dysregulation is a key upstream event driving this signaling suppression. The mechanism may involve two aspects: direct inhibition of CaMKII autophosphorylation due to reduced cytosolic Ca^2+^ levels, and diminished troponin I phosphorylation (Figures 4D,E), which might impair the scaffolding or allosteric activation of CaMKII at the sarcomere. Consequently, HDAC4 hypophosphorylation is expected to promote its nuclear accumulation. Nuclear accumulation of HDAC4 is known to repress transcription of genes important for cardiac relaxation, such as KLF4 and SERCA2a. Thus, it is hypothesized that this signaling cascade represents a potential mechanistic link between the TPM1-E181K mutation and the diastolic impairment characteristic of RCM (Figures 4F,G).

The α-tropomyosin encoded by *TPM1* is a core regulatory protein for cardiac sarcomeric structure and function, coordinating calcium-dependent contraction-relaxation cycles through synergistic interactions with actin and the troponin complex (Keyt et al., 2022). While TPM1 mutations are predominantly reported in hypertrophic (HCM) and dilated cardiomyopathy (DCM)—attributed to sarcomeric hypercontractility (HCM) or actin destabilization (DCM) (Perry, 2001; Halder et al., 2024)—and while the E181K mutant was shown to cause hypercontractility *in vitro* by (Gupte et al., 2015), our study in a clinical RCM context reveals that its unique pathogenicity is mediated specifically through calcium-CaMKII-HDAC4 signaling axis disruption. This study further demonstrates that the E181K mutation, though not altering TPM1 expression levels (Figure 3C), impairs troponin phosphorylation (Figures 4D,E), and significantly reduces calcium affinity and diminishes intracellular Ca^2+^ transient amplitude (Figures 3E,F) This phenotype fundamentally differs from the RCM caused by compound heterozygous TPM1 mutations (E62Q/M281T) as reported by Dorsch et al. (Mazzarotto et al., 2020), In their report, E62Q (N-terminal domain) induces sarcomeric hypercontractility *via* disrupted actin binding, whereas E181K (central functional domain/Tropomyosin core) primarily disrupts calcium signaling rather than sarcomeric stability (Table 1). These findings demonstrate that mutations in distinct TPM1 domains drive phenotypic heterogeneity through divergent molecular mechanisms, suggesting unique signaling networks underlie RCM pathogenesis.

**TABLE 1:** Clinico-molecular characteristics of *TPM1* mutations in restrictive cardiomyopathy (RCM) (Dorsch et al., 2021).

Mutation	Domain location	Exon	Phenotype	Amino acid change	Pathogenic mechanism
c.184G > C	N-terminal stability (1-80aa)	2	Hypertrophic/RCM	Glu62Gln	Impaired actin binding → sarcomeric hypercontractility
c.842 T > C	C-terminal tail (∼Stop codon)	9		Met281Thr	Tropomyosin destabilization → sarcomeric structural disruption
c.541G>A	Central functional domain (tropomyosin core)	5	RCM	Glu181Lys	Calcium dysregulation → CaMKII/HDAC4 suppression

**FIGURE 5:**
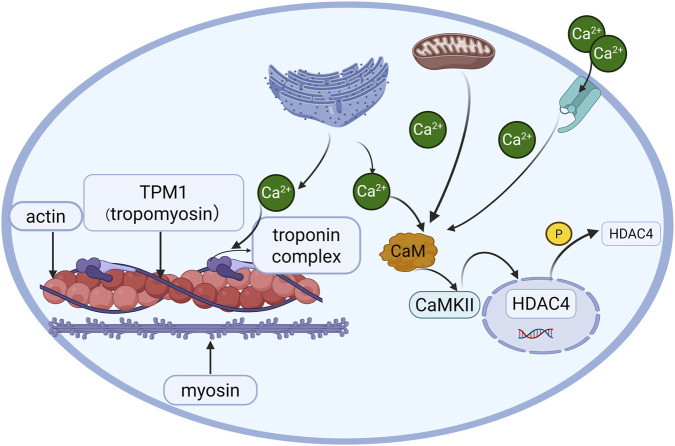
Proposed mechanism of the *TPM1*-p.E181K mutation in troponin and CaMKII/HDAC4 signaling.

Current therapeutic strategies for restrictive cardiomyopathy (RCM) primarily adhere to general heart failure management principles (Cimiotti et al., 2021), including angiotensin-converting enzyme inhibitors, β-blockers, calcium channel blockers, and anticoagulation. However, these approaches lack etiology-specific interventions. Based on our findings, targeted activation of CaMKII or inhibition of HDAC4 nuclear translocation emerges as a potential therapeutic avenue (Figure 5). However, it is important to note that this study did not functionally evaluate the reversal of pathological phenotypes through such modulation, which represents a key limitation and a necessary next step to validate these targets.

In summary, this study first establishes the association between the TPM1-p.E181K mutation and sporadic RCM, demonstrating that its pathogenesis is mediated through a cascade of events: calcium dyshomeostasis leads to suppression of CaMKII/HDAC4 phosphorylation, which subsequently causes sarcomeric disruption, and ultimately results in myocardial hypercontractility. This mechanistic insight provides novel therapeutic perspectives for RCM. Future studies investigating interventions targeting this pathway will be crucial to assess their potential in overcoming current treatment limitations.

## Data Availability

The original contributions presented in the study are included in the article/supplementary material, further inquiries can be directed to the corresponding authors.
